# Potential Interest of *Oxalis pes-caprae* L., a Wild Edible Plant, for the Food and Pharmaceutical Industries

**DOI:** 10.3390/foods13060858

**Published:** 2024-03-12

**Authors:** Jesús Clemente-Villalba, Francisco Burló, Francisca Hernández, Ángel A. Carbonell-Barrachina

**Affiliations:** 1 Research Group Food Quality and Safety, Instituto de Investigación e Innovación Agroalimentaria y Agroambiental (CIAGRO-UMH), Universidad Miguel Hernández de Elche (UMH), Ctra. Beniel, km 3.2, 03312 Orihuela, Alicante, Spain; jesus.clemente@goumh.umh.es (J.C.-V.); francisco.burlo@umh.es (F.B.); 2 Grupo de Investigación en Fruticultura y Técnicas de Producción, Instituto de Investigación e Innovación Agroalimentaria y Agroambiental (CIAGRO-UMH), Universidad Miguel Hernández de Elche (UMH), Ctra. Beniel, km 3.2, 03312 Orihuela, Alicante, Spain; francisca.hernandez@umh.es

**Keywords:** amino acids, fatty acids, minerals, sugars, volatile profile

## Abstract

(1) Background: *Oxalis pes-caprae* L. is a plant considered within the group of so-called Wild Edible Plants (WEPs). The particularity of these plants is that they grow only with the natural resources at their disposal. Unfortunately, these types of plants are undervalued, being regularly uprooted from the fields. (2) Methods: Therefore, this study aimed to valorize the *Oxalis pes-caprae* plant, analyzing the proximate composition (sugars, organic acids, minerals, amino acids profile, fatty acids content, and volatile profile) of the plant shoots (flower, leaves, and stem) to demonstrate the full potential of this WEP. (3) Results: The results showed that *Oxalis pes-caprae* can be considered a natural source of minerals; furthermore, 19 essential and non-essential amino acids were found. Regarding the fatty acid profile, flowers are an important source of linoleic acid, and leaves present a high amount of α-linolenic acid. (4) Conclusions: Therefore, this research provides new information that reaffirms the capacity of *Oxalis pes-caprae* L. (WEP) to be a plant with great future progression due to its nutritional quality since it could be used in the food, nutritional, or pharmaceutical fields. Further research must be conducted to assay the biomass production and the costs of recommending farmers not to destroy this plant in their fields.

## 1. Introduction

It is estimated that in 2050, the world’s population will be approximately 9 billion people; this increase will create a global problem linked to the lack of enough food to feed the entire population [1]. This fact together with the health problems associated with food (e.g., obesity, allergies, etc.), are challenges that the world will face in the future, although this last problem has already started. In the 1970s, the first warnings about harmful diets with a high content of sugars and/or too high fat intake, were reported. However, it was not until the 1990s when these tendencies normally linked to sedentarism began to show their negative effects on health, such as diabetes, hypertension, or obesity [2]. These consumption trends can also have a significant impact on the environment; for instance, by 2050, if these diets are not controlled, they will result in an 80% increase in greenhouse gas emissions [3].

The homogenization of diets and the standardization of crops (basically looking for high yields) have led to 94 cultivated species representing 90% of the world’s food supply, although only 9 represent 66% of total crop production [4,5]. Furthermore, currently, there are 866 domesticated species; therefore, wild edible plants (WEPs) could be used to further expand the number of species used to produce global food [5,6]. It must be considered that this action could also help the Sustainable Development Goals (SDGs), specifically numbers 2 and 12. Goal number 2 is “Zero Hunger” and focuses mainly on ending world hunger, achieving food security and improving nutrition, and promoting sustainable agriculture by 2030; in this sense, wild edible plants (WEPs) will be ideal for combating these inequalities [7]. In addition, SDG 12, “Responsible Production and Consumption”, evaluates the impact on resources such as water, energy, and food, exposing the production phase (agriculture) as one of the most serious stages with an environmental impact. The concept of WEP comes from the term wild plants described in 1999, which refers to plants that grow spontaneously without direct human intervention [8]. The families with the highest number of WEP species are Asteraceae, Brassicaceae, Fabaceae, Portulacaceae, and Oxalidaceae, among others [9,10].

The WEP *Oxalis pes-caprae* (Oxalidaceae), commonly called “sourgrass” or “vinagrillo o agrio” (in Spanish), normally grows in the areas of Mediterranean or subtropical climates; although this plant is native to South Africa [11], and it is an invasive species. This plant is a bulbiferous geophyte with pentameric and pentacyclic flowers, with five sepals and five petals fused at the base [12]. Regarding the properties of Oxalis pes-caprae, several studies reported its antioxidant (mainly due to the action of polyphenols) and anti-inflammatory properties, cytotoxic and phytotoxic activity, possible neuroprotective effects, antibacterial, antifungal activity and inhibition of alpha-amylase and alpha-glucosidase; therefore, it seems quite reasonable to think that this plant can be considered an interesting natural source of phytochemicals with potential application in pharmacological applications [13,14,15,16,17,18,19]. To our knowledge, no studies have been carried out about the content of amino acids, sugars, volatile composition, or even the proximal composition of the different parts (flowers, leaves, and stems) of *Oxalis pes-caprae* L. Consequently, the aim of this study was to determine the nutritional and chemical composition of the different parts of the *Oxalis pes-caprae* L. plant. This information will provide a basis for the selection of the most suitable WEPs for use as a functional ingredient and to be able to develop new food products in particular. It would determine whether the aerial portions of this WEP are of interest for further domestication and/or use in the food or pharmaceutical industries.

## 2. Materials and Methods

### 2.1. Plant Material

*Oxalis pes-caprae* plants used were collected at the Orihuela campus of the Universidad Miguel Hernández de Elche (38°4′10″ N, 0°59′1″ O, Alicante, Spain) in February 2022. Harvested plants of *Oxalis pes-caprae* were washed with tap and distilled water and separated into flowers, leaves, and stems. Samples were lyophilized before storage at room temperature.

### 2.2. Proximate Characterization

In the three parts (flowers, leaves, and stems) of *Oxalis pes-caprae*, the following parameters were analyzed: moisture, ash, total dietary fiber, fat, and protein (Kjeldahl method using a conversion factor of 6.25) according to the AOAC [20]. Available carbohydrates were calculated using the formula:

Carbohydrates (%) = 100 − (% moisture + % ash + % fat + % protein)

while the energy or total caloric value (kcal) was determined by the formula on the basis of a 100 g portion [20]:

Energy (kJ) = [(% protein × 4 kcal/g) + (% carbohydrates × 4 kcal/g) + (% fat × 9 kcal/g)] × 4184

### 2.3. Analysis of Sugars, Organic Acids

Sugars and organic acids were quantified according to Hernández et al. [21] with some modifications using 0.150 g of sample. The determination of the content of sugars and organic acids was conducted using high-performance liquid chromatography (HPLC–DAD-RID) (Hewlett Packard 1100 series; Willmington, DE, USA). A Supelcogel TM C-610H column (30 cm × 7.8 mm) and a Supelguard precolumn (5 cm × 4.6 mm) (Supelco, Bellefonte, PA, USA) were used for separation. The absorbance was measured using a diode-array detector (DAD) at 210 nm for the organic acids detection and a refractive index detector (RID) was used for the detection of sugars. Standards of organic acids (citric, fumaric, malic, oxalic, phytic, and tartaric) and sugars (arabinose, fructose, galactose, glucose, maltose, and sucrose) were obtained from Sigma (St. Louis, MO, USA). Calibration curves, with a concentration range between 1 and 10 g L^−1^, were used for the quantification of organic acids and sugars and showed good linearity (r^2^ ≥ 0.999). This analysis was run in triplicate, and the results were expressed as g kg^−1^.

### 2.4. Analysis of Minerals and Ascorbic Acid (Vitamin C)

The determination of minerals was carried out according to Cerdá-Bernad et al. [22] using ~0.100 g of freeze-dried samples. Total concentrations of macronutrients (Ca, Mg, Na, and K) and micronutrients (Zn, Cu, Mn, and Fe) in the previously mineralized samples were quantified with an Inductively Coupled Plasma Mass Spectrometer (ICPMS-2030, Shimadzu, Kyoto, Japan). 

For the extraction of the ascorbic acid, ~50 mg of the lyophilized samples were weighed and dissolved in 1 mL of extractant MeOH:H_2_O:HCOOH (75:24:1) (*v*/*v*/*v*) using an ultrasonic bath for short periods of 2–3 min. The samples were then centrifuged at 12,000× *g* for 15 min and filtered using a filter of 0.22 µm. For the analysis of the samples, the liquid chromatography equipment UPLC-QToF-MS (Agilent, UPLC-QTOF 6550-I-Funnel, Santa Clara, CA, USA) was used, with the mobile phases: Mobile phase A: 0.5% aqueous formic acid, and Mobile phase B: Methanol/water (50:50, *v*/*v*) containing 0.5% formic acid.

### 2.5. Analysis Amino Acids

Amino acids were quantified according to Kıvrak et al. [23] with some modifications. Approximately 100 mg of each sample was placed into a tube containing 1 mL of 0.1% (*v*/*v*) formic acid in water–methanol (80:20) (*v*/*v*) solution. Then, the sample was injected into a UPLC-QToF-MS (Agilent, UPLC-QTOF 6550-I-Funnel, Santa Clara, CA, USA) with the same mobile phases as for the analysis of ascorbic acid. All standards (arginine, alanine, asparagine, aspartic acid, cysteine, cystine, glutamine, glutamic acid, glycine, histidine, isoleucine, serine, proline, valine, threonine, leucine, lysine, methionine, phenylalanine, tyrosine, and tryptophan) were purchased from Sigma–Aldrich (St. Louis, MO, USA). Stock standard solutions of amino acids (1000 mg L^−1^) were prepared in distilled water.

### 2.6. Analysis Fatty Acids

Fatty acids were quantified according to Park and Goins [24] with some modifications. Approximately ~0.100 g of freeze-dried sample was weighed and placed into a test tube; then, 100 μL of dichloromethane and 1 mL of 0.5 N NaOH in methanol were added, and the test tube was closed and placed in a hot water bath at 90 °C for 10 min. Then, the test tube was rapidly cooled in an ice bath for 3 min. One mL of BF3 in methanol was added, and the test tube was placed in the dark for 30 min; later, 1 mL of ultrapure water and 600 μL of hexane were added. The sample was vigorously shaken for 1 min in a vortex (VORTEX 1, IKA, Staufen, Germany) and immediately afterward, centrifuged at 4000 rpm for 10 min (Eppendorf 5804R, Eppendorf, Hamburg, Germany). Subsequently, the supernatant was carefully recovered and placed into an amber chromatography vial. 

For separation, a gas chromatograph (GC) Shimadzu GC-2030 coupled with a flame ionization detector (FID) with an automatic injector AOC-20i was used (Shimadzu Scientific Instruments, Inc., Columbia, MD, USA). Helium was used as a carrier gas, and nitrogen was used as a make-up gas (24 mL min^−1^). FID used hydrogen and air at rates of 32 mL min^−1^ and 200 mL min^−1^, respectively. The GC system used a Supelco SP^®^-2380 capillary column (60 m × 0.25 mm × 0.20 μm) (St. Louis, MO, USA). The detector temperature was kept at 260 °C, and a 1:20 split ratio and a total lineal flow velocity of 28.4 cm s^−1^ were used. The oven temperature started at 70 °C and increased up to 250 °C at a rate of 3 °C min^−1^. Methyl fatty acids were identified by comparison with the retention times of the FAME Supelco MIX-37 standards (Supelco Company, Bellefonte, PA, USA). Results were calculated as a percentage of each fatty acid in the total fatty acids profile.

### 2.7. Volatile Profile

Volatile compounds were quantified according to Noguera-Artiaga et al. [25] with some modifications. Between 0.150–0.300 g of freeze-dried samples were weighed and placed into a 40 mL vial with polypropylene cap and PTFE/silicone septa; isoamyl acetate (1000 mg/L) (5 μL) was added as the internal standard for semi-quantification of compounds. After 5 min at 45 °C (equilibration time), a 50/30 μm DVB/CAR/PDMS fiber was exposed to the vial headspace at 40 °C with continuous agitation (250 rpm) in a magnetic stirrer (IKA C-MAG HS 4, IKA-Werke GmbH & Co. KG, Staufen, Germany). After 45 min of exposure, the fiber was extracted from the vial and placed into the GC-MS injector. The separation and identification of compounds was performed using a Shimadzu GC-MS Nexis GC2030 (Shimadzu Scientific Instruments, Inc., Columbia, MD, USA), equipped with a Sapiens X5MS column (30 m × 0.25 mm × 0.25 μm) (Teknokroma, Barcelona, Spain), and coupled with a mass spectrometer detector (TQ8040 NX triple quadrupole mass spectrometer; Shimadzu Scientific Instruments, Inc., Columbia, MD, USA). Only the single quadrupole acquisition mode was used on the TQ8040 NX (Q3 Scan; scan speed 5000 amu s^−1^; mass range 40–400 *m/z*; event time 0.100 s). The oven temperature program was as follows: (i) initial temperature of 35 °C, hold for 5 min; (ii) increment of 5 °C min^−1^ up to 150 °C, hold for 1 min; (iii) increment of 10 °C min^−1^ up to 280 °C and hold for 5 min. Helium column head pressure was 47.6 kPa (constant linear velocity mode of 36 cm s^−1^). The injector, ion source, and interface were at 250, 230, and 280 °C, respectively. Helium was used as the carrier gas, column flow of 1 mL min^−1^, with a split ratio of 1:50, and a purge flow of 6 mL min^−1^.

Retention indexes of a commercial alkane standard mixture (Sigma-Aldrich, Steinheim, Germany) were used to identify the compounds, as well as the NIST 17 Mass Spectral and Retention Index Libraries. The identification was considered tentative when it was based only on mass spectral data, and only compounds with spectra similarity > 90% were considered correct hits. The linear retention similarity filter was set at ±10 units.

### 2.8. Statistical Analysis

In general, experimental data were subjected first to one-way analysis of variance (ANOVA) and later to Tukey’s multiple range test to compare the means. Differences were considered statistically significant at *p* < 0.05. All statistical analyses were performed using StatGraphics Plus 5.0 software (Manugistics. Inc., Rockville, MD, USA).

## 3. Results and Discussion

### 3.1. Proximate Composition

The proximate composition (moisture, ash, total dietary fiber, protein, fat, and carbohydrates) was significantly affected by the part of the plant under study (Table 1), except energy.

Regarding moisture, the stems had the highest value in both the fresh and freeze-dried samples, with values of 11.10 and 90.95%, respectively. These values are far higher than those reported by Datta et al. (2019), 60.28%, in similar plants such as *Oldenlandia corymbosa*. However, in most of the previous studies, the moisture varied in the range of 80–95% moisture [26,27,28].

The ash content showed a completely different behavior from that of moisture, with leaves showing the highest value (12.93%) as compared to flowers (8.27%) and stems (3.15%). The ash content found in the *Oxalis pes-caprae* leaves is significantly higher than those reported in *Oxalis corymbosa* (3.91%) by Vera, Espino Manzano, and Hernandez [19]. Ash values obtained in *Oxalis pes-caprae* flowers were similar to those reported by Datta et al. [29] in *Oldenlandia corymbosa* (8.34%). However, a study conducted in Pakistan shows much higher ash results (28.00%) therefore the geographical influence on the results is important [17].

Regarding the total dietary fiber, the highest content was obtained in the stems (36.36%) of *Oxalis pes-caprae* followed by the flowers (30.68%) and leaves (28.72%). Current results highlight the fact that *Oxalis pes-caprae* has higher fiber content than other similar plants, such as *Oxalis tuberosa* (0.87–1.69%) and *Oldenlandia corymbosa* (7.26%) [29,30]. 

On the other hand, for both protein and fat content, *Oxalis pes-caprae* leaves showed the highest content, while stems showed the lowest content for both variables. Very similar protein contents were obtained for *Oldenlandia corymbosa* (10.52%) [29]. Another study on *Oxalis corniculata* leaves reported a total nitrogen content of 3.56%, which is equivalent to 22.25% [31] after the application of a 6.25 conversion factor [20]; thus, this value is close to that found in the leaves of *Oxalis pes-caprae*. Regarding fat, the leaves had 12.68%, while the stems only had 3.53%. These fat values were lower than those found previously reported in *Oxalis corniculata*, 23.75% [31], although in this study, the same plants collected in different areas had protein contents ranging between 13.4–17.6%. The percentage of carbohydrates in the parts of *Oxalis pes-caprae* indicated a higher value in the stems (73.34%), followed by flowers (62.57%) and leaves (45.87%); these values were higher than those found by Jain et al. (2010) in *Oxalis corniculate* (24.67%) but similar to those previously reported in plants of other genera such as Malvaceae and Lamiaceae, 75–85% [31,32,33]. Finally, the total energy contents were equivalent in the three parts of the *Oxalis pes-caprae* plant. These values are quite high as those previously reported for plants of similar genera, such as Fabaceae, Malvaceae, and Lamiaceae, ~1548–1694 kJ [32,33,34].

### 3.2. Sugars, Organic Acids and Ascorbic Acid (Vitamin C)

The contents of fructose, sucrose, and oxalic acid were statistically equivalent in the flowers, leaves, and stems of *Oxalis pes-caprae* (Table 2). 

The leaves of *Oxalis pes-caprae* were richer in maltose than the rest of the plant, while they had the lowest content of glucose; this sugar (glucose) was more abundant in stems. The fructose in *Oxalis pes-caprae* was higher than those found in other plants such as *Calligonum comosum* L. or *Cynara cardunculus* L. [35,36]; however, in the flowers of *Moringa oleifera* Lam. and *Malva sylvestris* L., 75.6 and 87.2 g kg^−1^, respectively, [32,37] the values were very similar to those found in the current study. The contents of glucose found here were close to those previously reported in *Cynara cardunculus* L., *Moringa oleifera* Lam., or *Malva sylvestris* L., 99.5, 120.7, 73.6 g kg^−1^, respectively [32,36,37]. Finally, it is important to comment that sucrose is the predominant sugar in most WEPs [35,36,38,39]; however, the values of sucrose found in these studies were lower than those reported here for *Oxalis pes-caprae*.

The concentration of oxalic acid found in *Oxalis pes-caprae* L. is much higher than those found in other types of WEPs, such as *Allium ampeloprasum* L. (27.83 mg/100 g) [26]. However, the current values were closer to those found in the leaves of *Cynara cardunculus* L. var. *altilis* (81 g kg^−1^) [36]. These high values of oxalic acid were expected because the genus *Oxalis* took the name after the high content of oxalic acid found in these plants. Oxalic acid and its salts, called oxalates, can cause problems in the human body because they scavenge minerals such as calcium, although it has been calculated that the problems would arise above 150 mg of daily intake of oxalates [40]. 

On the other hand, the ascorbic acid accumulated mainly in the stems and leaves of *Oxalis* plants, and significantly less in the flowers (Table 2). The content of this organic acid was higher in other studied WEPs, such as *Blumea lacera* (127 mg/100 g), *Commelina benghalensis* (23.6 mg/100 g), or even *Dioscorea praehensilis* with 10 mg/100 g [41,42,43]. In plants belonging to the same family, another study carried out by Šircelj et al. [44] with *Oxalis acetosella* showed much higher values (3457 μg/g dw) than those found in *Oxalis pes-caprae*.

### 3.3. Minerals

The mineral composition found in *Oxalis pes-caprae* is shown in Table 3, with potassium and Fe predominating among the macro- and micro-nutrients, respectively.

Calcium (Ca) concentration was higher in the stems (620 mg/100 g), and is six times lower in the flowers (104 mg/100 g). A study conducted by Datta, Sinha, Bhattacharjee, and Seal [29] on six WEPs showed Ca contents ranging from 492 to 621 mg/100 g; this range is very close to that found in *Oxalis* leaves and stems. Regarding potassium (P), stems (1399 mg/100 g) and flowers (1247 mg/100 g) showed similar contents and were significantly higher than leaves. K content found here was higher than those reported in *Enhydra fluctuans* (487 mg/100 g) but much lower than those reported in other 19 WEPs, reaching a value as high as 7830 mg/100 g in *Smyrnium cordifolium* Boiss [45]. Regarding sodium (Na), there were no differences between the contents of leaves and stems, but they were higher than the Na content in the flowers. *Cichorium intybus* L. showed a higher sodium concentration (80.61 mg/100 g) in a study carried out by Jalali and Fakhri [45]. The *Oxalis* flowers had similar Na contents to those of other types of plants, such as *Allium hirtifolium* Boiss., *Stachys lavandulifolia* Vahl, or *Taraxacum vulgar* Hodn. Mzt. (30.28, 30.26, 30.35 mg/100 g, respectively) [45]. The content of magnesium (Mg) followed the order leaves > flowers > stems, with these contents being similar to those previously found in *Anchusa italica* Retz (120 mg/100 g) [45]. 

The most abundant micro-nutrient was Fe, especially in the flowers. No biologically significant differences were observed in the contents of Mn and Zn. The experimental contents of these three nutrients are similar to those previously reported in other WEPs [45]. 

### 3.4. Amino Acids

Nineteen amino acids were found in *Oxalis pes-caprae* L. (Table 4). In general, the essential amino acids predominated in the flowers, followed by leaves and, finally, stems, with leucine, isoleucine, and valine being the most abundant compounds of this chemical family. In this way, leucine also predominates in other plants [18,46,47]. These three amino acids (leucine, isoleucine, and valine) have an important function in plants by contributing to the volatile compounds responsible for their odor and aroma. These volatiles produced by plants not only have aromatic functions but can also act as aromas that attract pollinators; therefore, their role is fundamental [48]. 

Regarding the non-essential amino acids, the contents of flowers and leaves were similar and higher than those of the stems, with alanine and glutamic acid being the predominant compounds. However, alanine was the most abundant compound in the stems, while glutamic acid predominated in the leaves. In other WEPs such as *Sesamum indicum* L., *Balanites aegyptiaca* (L.) Delile [46] and *Portulaca oleracea* L. [18], the most abundant compound was glutamic acid. Glutamic acid is one of the four main ligands of zinc; this mineral performs catalytic or structural functions in plants [49].

### 3.5. Fatty Acids

In the present study, 29 fatty acid methyl esters (FAMEs) were identified (Table 5) in different tissues of *Oxalis pes-caprae*; these FAMEs consisted of are composed of 8 monounsaturated fatty acids (MUFAs), 7 polyunsaturated fatty acids (PUFAs) and 14 saturated fatty acids (SFAs). Although the number of SFAs was higher, the unsaturated FAMEs predominated and represented as much as 70–80% of the total content: flowers (MUFA + PUFA) = 73.72%; leaves (MUFA + PUFA) = 80.37%; and stems (MUFA + PUFA) = 77.76%.

The three most abundant compounds were C18:3n3 (-linolenic), C18:2n6c (linoleic), and C16:0 (palmitic). In previous studies on wild edible plants, these three same compounds, along with oleic acid, were the predominant ones [14,50,51]. Regarding linolenic acid (FA27), there were significant differences among the tissues under analysis, with leaves having the highest content (53.57%). These results agree well with previous studies reporting contents of ~50% of linolenic acid [27,52,53]; this content was reported in Amaranthaceae, Asteraceae, Montiaceae, Polygonaceae and Caryophyllaceae plants and more precisely in *Beta maritime* (57.80%), *Chondrilla juncea* (56.27%), *Montia fontana* (55.57%), *Rumex acetosella* (51.34%), *Rumex induratus* (58.84%), and *Silene vulgaris* (54.5%). However, the presence of linoleic acid (C18:2n6c, FA23) was higher in the flowers, reaching 47.65%. Other WEPs with a similar content of these compounds were *Allium ampeloprasum* (53.45%) and *Tamus communis* (42%), belonging to the Amaryllidaceae and Dioscoreaceae families, respectively [26,39]. 

The third major fatty acid was palmitic acid (FA5) with a presence of 18.51% in flowers. Regarding palmitic acid, several WEPs showed similar results, as is the case of *Diplotaxis erucoides* (18.23%) or *Humulus lupulus* (19.52%), belonging to the Brassicaceae and Cannabaceae families, respectively [53,54]. Oleic acid (FA19) predominated in the stems, followed by leaves and flowers. Oleic acid was present in other WEPs with values similar to those found in the stem of *Oxalis*. *Chenopodium ambrosioides*, *Helichrysum stoechas,* and *Scolymus hispanicus* have between 6 and 7% oleic acid content [32,38,53]. However, plants belonging to the lamiaceae family showed a higher content of oleic acid compared to other families. *Glechoma hederacea*, *Thymus pulegioides*, and *Thymus mastichina* showed 35.12%, 11.50%, and 9.82%, respectively [33,55].

**Table 5:** Fatty acid profile (main groups and ratios) of *Oxalis pes-caprae* L.

Code	FA (%) ^ϒ^	R. Time	ANOVA ^†^	Flower	Leaf	Stem
FA1	C12:0 (Lauric)	19.330	***	0.31 ± 0.06 a ^‡^	0.09 ± 0.05 b	0.08 ± 0.019 b
FA2	C13:0 (Tridecanoic)	21.419	-	n.d.	n.d.	0.02 ± 0.002
FA3	C14:0 (Myristic)	23.420	***	0.51 ± 0.06 a	0.31 ± 0.02 b	0.20 ± 0.08 c
FA4	C15:0 (Pentadecanoic)	25.348	***	0.06 ± 0.001 a	0.02 ± 0.001 b	0.06 ± 0.004 a
FA5	C16:0 (Palmitic)	27.251	***	18.51 ± 0.47 a	11.73 ± 0.74 c	16.42 ± 0.43 b
FA6	C17:0 (Isomargaric)	28.156	***	0.02 ± 0.001 b	n.d.	1.41 ± 0.32 a
FA7	C17:0 (Margaric)	28.943	***	0.13 ± 0.006 a	0.10 ± 0.005 b	0.11 ± 0.01 b
FA8	C18:0 (Stearic)	30.649	***	2.05 ± 0.11 a	1.22 ± 0.07 c	1.46 ± 0.01 b
FA9	C19:0 (Nonadecanoic)	32.318	-	n.d	n.d.	0.18 ± 0.009
FA10	C20:0 (Arachidic)	33.923	***	0.09 ± 0.005 c	0.24 ± 0.03 a	0.16 ± 0.04 b
FA11	C21:0 (Heneicosanoic)	35.336	***	0.15 ± 0.01 a	0.15 ± 0.05 a	0.04 ± 0.003 b
FA12	C22:0 (Behenic)	36.831	***	1.33 ± 0.30 a	1.01 ± 0.20 b	0.01 ± 0.0001 c
FA13	C23:0 (Tricosylic)	38.165	***	0.97 ± 0.20 b	2.87 ± 0.76 a	0.51 ± 0.44 c
FA14	C24:0 (Lignoceric)	39.585	***	0.59 ± 0.04 b	0.07 ± 0.01 c	0.92 ± 0.07 a
	**Σ SFA**		***	**24.72 a**	**17.81 c**	**21.58 b**
FA15	C15:1 (Pentadecenoic)	26.835	***	0.06 ± 0.003 b	0.16 ± 0.01 a	0.07 ± 0.006 b
FA16	C16:1 (Palmitoleic)	27.856	-	0.01	n.d.	n.d.
FA17	C16:1c9 (Hypogeic)	28.275	***	0.12 ± 0.002 b	n.d.	0.89 ± 0.09 a
FA18	C18:1t9 (Elaidic)	31.316	***	0.36 ± 0.009 c	8.90 ± 0.45 a	4.05 ± 0.05 b
FA19	C18:1c9 (Oleic)	31.517	***	0.76 ± 0.009 c	1.43 ± 0.09 b	6.59 ± 0.74 c
FA20	C18:1n7 (*cis*-Vaccenic)	31.660	***	0.30 ± 0.008 c	0.42 ± 0.01 b	0.67 ± 0.009 a
FA21	C22:1n9 (Erucic)	37.730	***	1.22 ± 0.26 a	0.02 ± 0.001 b	n.d.
FA22	C24:1n9 (Nervonic)	40.122	***	0.04 ± 0.007 c	0.08 ± 0.01 b	0.18 ± 0.07 a
	**Σ MUFA**		***	**2.87 c**	**11.01 b**	**12.45 a**
FA23	C18:2n6c (Linoleic)	32.941	***	47.65 ± 0.37 a	10.15 ± 0.56 c	29.57 ± 1.44 b
FA24	C20:2 (Eicosadienoic)	35.998	***	2.77 ± 0.04 a	0.32 ± 0.04 c	0.87 ± 0.04 b
FA25	C22:2 (Docosadienoic)	38.712	***	1.47 ± 0.59 b	2.95 ± 0.37 a	n.d.
	**Σ n-6 PUFA**		***	**51.89 a**	**13.42 c**	**30.44 b**
FA26	C18:3n6 (γ-Linolenic)	33.849	***	0.58 ± 0.03 b	1.20 ± 0.07 a	0.42 ± 0.07 c
FA27	C18:3n3 (α-Linolenic)	34.552	***	18.17 ± 0.03 c	53.57 ± 2.45 a	34.04 ± 2.05 b
FA28	C20:3n3 (Eicosatrienoic)	36.894	***	0.10 ± 0.06 b	1.08 ± 0.13 a	0.03 ± 0.02 b
FA29	C20:3n6 (dihomo-γ-Linoleic)	37.497	***	0.11 ± 0.007 b	0.09 ± 0.008 b	0.38 ± 0.02 a
	**Σ n-3 PUFA**		***	**18.96 c**	**55.94 a**	**34.87 b**
	**Σ PUFA**		**NS**	**70.85**	**69.36**	**65.31**

^†^ *** significant at *p* < 0.001. ^‡^ Values (mean of 3 replications) followed by the same letter within the same row were not significantly different (*p* > 0.05) according to Tukey’s least significant difference test. ^ϒ^ FA = Fatty acid; SFA: saturated fatty acids; MUFA: monounsaturated fatty acids; PUFA: polyunsaturated fatty acids. n.d.: not detected. NS: Not Significant.

### 3.6. Volatile Profile

A total of 32 volatile compounds were isolated, identified, and quantified in the aerial tissues of *Oxalis pes-caprae*, as well as all the aromatic descriptors associated with the volatile compounds (Table 6) [56,57,58,59,60,61]. The volatile compounds can be grouped into six chemical families: esters (11 compounds), alkanes (7), terpenes (5), alcohols (5), aldehydes (2), acids (1), and ethers (1). Fifteen out of the 32 volatile compounds were found to be significantly different amounts in the studied tissues of this plant. The three main compounds found were nerolidol, caryophyllene, and 3-hexen-1-ol acetate.

In general, nerolidol (V27) was the compound with the highest concentration (21.58 μg/g) in all the tissues of this plant, more precisely in the flowers. Nerolidol is typically associated with a floral odor and is present mainly in the essential oils of flowers. The presence of this compound in flower is very important because it has been associated with antifungal, antibacterial, and antioxidant potential [16,62]. Regarding β-caryophyllene (V20) was also found exclusively in the flowers, and its sensory descriptors are sweet, woody, spicy, and clove. Humulene (α-caryophyllene), α-terpineol or β-farnesene (1.58, 1.13, and 3.51 μg/g, respectively) were also found but in lower concentrations, demonstrating the importance of the terpenes group in the volatile profile of this particular WEP. This statement was also supported by the results Fukalova Fukalova et al. [63], with β-caryophyllene being found in six out of the seven plants studied, with Porophyllum ruderale showing the highest content of this compound. 

3-Hexenyl acetate (V7) was found in all three parts of the plant, with the highest content being found in the leaves (16.0 μg/g). This compound was also present in carrot leaves, parsley, and the Nelumbo Nucifera flowers belonging to the Nelumbonaceae family [64,65]. The content of pentyl acetate (V4) was also of importance, with leaves and stems having significantly higher contents than flowers.

The total content of volatile compounds was significantly higher in the *Oxalis* flowers, followed by leaves and stems. This behavior is quite logical as normally flowers smell more intensely than leaves, and these more than stems.

## 4. Conclusions

Three parts of *Oxalis pes-caprae* L. (flowers, leaves, and stems) were analyzed to deepen their composition. The flower stood out in its sugar content, such as fructose and sucrose. Regarding minerals, it was the part of *Oxalis* that had the highest iron content, and the only one that had a zinc concentration. This part also stood out for having the highest concentration of amino acids in the entire plant. Apart from these values, the results obtained in the fatty acid profile were more than 50% in the Σ n-6 PUFA acids in the whole plant. In the leaves, its protein content stood out compared to the flowers and stems; also, it stood out in its concentration of maltose. Regarding minerals, the flowers stood out in their magnesium and manganese content. In the fatty acid profile, alpha-linolenic acid had the highest content compared to the rest of the acids. The stems showed in the proximal composition the highest total dietary fiber content, and the lowest fat value. It also showed the highest concentrations of calcium, potassium, and sodium. In the fatty acid profile, it was the most balanced, obtaining around 30% in both Σ n-6 PUFA and Σ n-3 PUFA. For the introduction of *Oxalis pes-caprae* as food, it should be domesticated, obtaining growing conditions that could allow lowering the levels of oxalic acid, and therefore, ingesting it directly without limitations. This fact is independent of taking advantage of the antibacterial, antifungal properties and cytotoxic inhibition capacity that the plant has. The interesting insight about the results shown by *Oxalis pes-caprae* L., is that all parts can be considered high-value biomass. This allows us to affirm that the plant is valid as a whole because each part of it adds a different type of nutritional contribution after its intake. The results make evident the promising future and the potential of this plant for industries such as agri-food or pharmaceutical, being an undervalued and discarded plant.

## Figures and Tables

**Table 1:** Proximate composition of *Oxalis pes-caprae* L.

Parameters (%)	Flowers	Leaves	Stems	*p*-Value	ANOVA ^†^
Moisture (freeze-dried)	9.05 ± 0.78 b ^‡^	9.17 ± 1.22 b	11.10 ± 0.54 a	0.0000	***
Moisture	85.86 ± 0.26 b	86.07 ± 0.36 b	90.95 ± 0.57 a	0.0000	***
Ash	8.27 ± 0.20 b	12.93 ± 0.72 a	3.15 ± 1.01 c	0.0000	***
Total dietary fiber	30.68 ± 1.32 b	28.72 ± 1.50 b	36.36 ± 3.69 a	0.0000	***
Protein	13.45 ± 0.28 b	19.35 ± 0.54 a	8.86 ± 1.78 c	0.0000	***
Fat	6.66 ± 2.16 b	12.68 ± 0.10 a	3.53 ± 0.40 c	0.0000	***
Carbohydrates	62.57 ± 0.55 b	45.87 ± 0.80 c	73.34 ± 1.15 a	0.0000	***
Energy (kJ)	1522 ± 16.90	1569 ± 23.04	1506 ± 18.57	0.1044	NS

^†^ NS: not significant at *p* > 0.05; *** significant at *p* < 0.001. ^‡^ Values (mean of 3 replications) followed by the same letter within the same row were not significantly different (*p* > 0.05) according to Tukey’s least significant difference test.

**Table 2:** Sugars and organic acids found in *Oxalis pes-caprae* L.

Compound (g kg^−1^ dw)	Flowers	Leaves	Stems	*p*-Value	ANOVA ^†^
Fructose	107 ± 33.77	103 ± 4.65	77.9 ± 8.90	0.2421	NS
Glucose	75.3 ± 18.59 a ^‡^	33.2 ± 3.43 b	88.9 ± 1.09 a	0.0019	**
Maltose	n.d.	40.35 ± 6.22 a	14.04 ± 1.87 b	0.0022	**
Sucrose	193 ± 14.76	192 ± 4.20	173 ± 3.03	0.0792	NS
Oxalic acid	98.0 ± 21.51	98.2 ± 6.55	110 ± 7.56	0.4855	NS
Ascorbic Acid (mg 100 g^−1^ dw)	0.42 ± 0.006 c	3.17 ± 0.007 b	3.50 ± 0.004 a	0.0000	***

^†^ NS: not significant at *p* > 0.05; ** significant at *p* < 0.01, *** significant at *p* < 0.001. ^‡^ Values (mean of 3 replications) followed by the same letter within the same row were not significantly different (*p* > 0.05) according to Tukey’s least significant difference test. n.d.: not detected.

**Table 3:** Mineral composition of *Oxalis Pes-caprae* L.

Mineral [mg (100 g)^−1^ dw]		Flowers	Leaves	Stems	*p*-Value	ANOVA ^†^
Macro	Ca	104 ± 16.97 c ^‡^	620 ± 20.21 b	453 ± 10.60 a	0.0000	***
	K	1247 ± 3.53 b	859 ± 20.56 c	1399 ± 21.21 a	0.0000	***
	Na	35.1 ± 12.47 b	69.1 ± 1.20 a	71.5 ± 3.88 a	0.0000	***
	Mg	95.4 ± 2.47 b	129 ± 2.80 a	73.2 ± 0.00 c	0.0000	***
Micro	Fe	7.7 ± 0.28 a	3.2 ± 0.16 b	1.4 ± 0.03 c	0.0000	***
	Mn	0.87 ± 0.00 b	1.18 ± 0.01 a	0.32 ± 0.00 c	0.0000	***
	Zn	0.30	n.d.	n.d.	-	-

^†^ *** significant at *p* < 0.001. ^‡^ Values (mean of 3 replications) followed by the same letter within the same row were not significantly different (*p* > 0.05) according to Tukey’s least significant difference test. n.d.: not detected.

**Table 4:** Amino acids found in *Oxalis pes-caprae* L.

Amino Acids [mg (100 g)^−1^ dw]		Flowers	Leaves	Stems	*p*-Value	ANOVA ^†^
Essential	Arginine	13.0 ± 0.37 a ^‡^	4.91 ± 0.02 b	2.04 ± 0.08 c	0.0000	***
	Histidine	19.9 ± 7.32 a	8.98 ± 0.03 b	5.57 ± 0.04 c	0.0000	***
	Isoleucine	209 ± 1.67 a	169 ± 3.45 b	170 ± 6.63 b	0.0000	***
	Leucine	306 ± 4.24 a	244 ± 9.65 b	245 ± 12.30 b	0.0000	***
	Lysine	5.46 ± 0.70 b	6.63 ± 0.14 a	2.16 ± 0.08 c	0.0000	***
	Methionine	6.01 ± 0.24 a	4.82 ± 0.08 b	1.83 ± 0.17 c	0.0000	***
	Phenylalanine	48.7 ± 3.20 a	50.6 ± 1.17 a	29.9 ± 2.60 b	0.0000	***
	Threonine	114 ± 3.49 a	13.9 ± 0.22 c	20.3 ± 0.25 b	0.0000	***
	Tryptophan	80.1 ± 6.07 a	47.2 ± 1.00 b	24.8 ± 3.91 c	0.0000	***
	Valine	187 ± 9.47 a	189 ± 7.02 a	134 ± 1.72 b	0.0000	***
Non-essential	Alanine	302 ± 12.44 b	307 ± 7.40 b	343 ± 11.17 a	0.0000	***
	Asparagine	n.d.	8.42 ± 0.22 a	5.94 ± 0.29 b	0.0000	***
	Aspartate	73.0 ± 3.27 b	91.1 ± 3.72 a	56.4 ± 6.03 c	0.0000	***
	Cysteine	0.55 ± 0.03 b	0.69 ± 0.02 a	n.d.	0.0000	***
	Glutamic acid	237 ± 11.93 b	325 ± 20.9 a	220 ± 4.57 c	0.0000	***
	Glycine	55.7 ± 6.84 a	11.9 ± 1.41 c	30.4 ± 4.72 b	0.0000	***
	Proline	102 ± 15.32 a	30.5 ± 0.30 c	50.7 ± 4.01 b	0.0000	***
	Serine	112 ± 22.49 a	46.3 ± 2.30 b	46.3 ± 1.42 b	0.0000	***
	Tyrosine	13.8 ± 0.82 c	40.1 ± 2.84 a	17.0 ± 0.80 b	0.0000	***
**TOTAL**		**1885 a**	**1600 b**	**1405 c**	**0.0000**	***

^†^ *** significant at *p* < 0.001. ^‡^ Values (mean of 3 replications) followed by the same letter within the same row were not significantly different (*p* > 0.05) according to Tukey’s least significant difference test. n.d.: not detected.

**Table 6:** Identification, concentration, and odor descriptors of volatile compound found in *Oxalis pes-caprae* L.

Code	Compound (μg g^−1^)	CF	RT (min)	KI (EXP)	KI (LIT)	Flowers	Leaves	Stems	ANOVA ^†^	Odor Descriptors ^¥^
V1	3-Methyl butanal	Aldehyde	3.349	690	686	3.02 ± 0.2 ^‡^ a	2.95 ± 0.1 a	0.08 ± 0.001 b	***	Aldehydic, fatty
V2	3-Hexen-1-ol	Alcohol	10.173	857	857	-	-	0.57 ± 0.06	-	Green, vegetable, herbal
V3	4-Penten-1-yl acetate	Ester	11.963	901	901	1.52 ± 0.42 a	1.39 ± 0.17 b	1.14 ± 0.24 b	***	Green, vegetable
V4	Pentyl acetate	Ester	12.434	914	917	4.33 ± 0.47 b	6.27 ± 0.35 a	6.50 ± 0.50 a	***	Fruity, banana
V5	Isoamyl propionate	Ester	14.537	970	969	0.98 ± 0.20 c	2.15 ± 0.33 a	1.52 ± 0.17 b	***	Sweet, fruity, banana
V6	Diisoamyl ether	Ether	15.728	1002	1002	2.03 ± 0.61 a	0.66 ± 0.06 b	0.36 ± 0.04 c	***	Fruity
V7	3-Hexenyl acetate	Ester	15.794	1004	1005	2.34 ± 0.30 c	16.0 ± 2.19 a	7.24 ± 1.16 b	***	Fresh, green, sweet, fruity
V8	Hexyl acetate	Ester	16.062	1011	1011	2.14 ± 0.16 c	2.64 ± 0.22 b	3.14 ± 0.29 a	***	Fruity, green, banana, sweet
V9	Pentyl butanoate	Ester	17.579	1056	1059	0.90 ± 0.08	-	-	-	Sweet, fruity, banana, cherry
V10	Linalool	Alcohol	19.021	1098	1098	3.19 ± 0.56	-	-	-	Floral, citrus, rose
V11	Nonanal	Aldehyde	19.178	1103	1102	0.95 ± 0.10	-	-	-	Waxy, aldehydic, citrus, fresh
V12	Isoamyl butanoate	Ester	19.231	1104	1104	0.95 ± 0.05	-	-	-	Sweet, fruity, green
V13	Phenylethyl alcohol	Alcohol	19.414	1110	1110	0.92 ± 0.13	-	-	-	Floral, rose
V14	α-Terpineol	Terpene	22.046	1194	1194	1.13 ± 0.21	-	-	-	Pine, lilac, woody, floral
V15	1,3-bis(1,1-dimethylethyl)benzene	Alkane	23.645	1248	1249	1.80 ± 0.25 a	0.26 ± 0.01 c	0.32 ± 0.007 b	***	-
V16	Nonanoic acid	Acid	24.005	1261	1267	1.59 ± 0.45	-	-	-	Waxy, dirty, cheese, dairy
V17	4,6-Dimethyl dodecane	Alkane	24.437	1275	1285	1.55 ± 0.02 b	1.64 ± 0.04 a	0.41 ± 0.01 c	***	Fruity, green
V18	1,1′-Bicyclohexyl	Alkane	25.708	1320	1307	2.70 ± 0.64	-	-	-	-
V19	Ethyl nonanoate	Ester	27.686	1290	1294	8.15 ± 1.67	-	-	-	Fruity, rose, waxy
V20	β-Caryophyllene	Terpene	28.528	1424	1424	19.86 ± 3.01	-	-	-	Sweet, woody, spicy, clove
V21	Isoamyl benzoate	Ester	28.915	1438	1437	1.58 ± 0.32	-	-	-	Sweet, balsamic, green, waxy
V22	β-Farnesene	Terpene	29.342	1454	1458	3.51 ± 0.89	-	-	-	Woody, citrus, herbal, sweet
V23	Humulene	Terpene	29.551	1462	1462	1.58 ± 0.44	-	-	-	Woody
V24	1-Dodecanol	Alcohol	29.887	1474	1474	1.49 ± 0.39 b	3.99 ± 0.85 a	0.77 ± 0.11 c	***	Earthy, soapy, waxy, fatty
V25	Pentadecane	Alkane	30.290	1490	1490	-	2.14 ± 0.57	-	-	Waxy
V26	2,4-bis(1,1-dimethylethyl)phenol	Alkane	30.642	1504	1502	1.34 ± 0.14 b	1.48 ± 0.06 a	0.46 ± 0.09 c	***	-
V27	Nerolidol	Terpene	31.826	1563	1562	21.58 ± 4.05	-	-	-	Floral, green, citrus
V28	Ethyl dodecanoate	Ester	32.412	1592	1591	3.92 ± 0.91 a	0.63 ± 0.14 b	-	***	Sweet, waxy, floral, soapy
V29	Hexadecane	Alkane	32.575	1600	1600	2.00 ± 0.31 a	0.84 ± 0.05 b	0.39 ± 0.02 c	***	Alkane
V30	Cyclotetradecane	Alkane	34.045	1691	1679	-	2.73 ± 0.77	-	-	Waxy
V31	1-Tetradecanol	Alcohol	34.149	1698	1686	-	3.06 ± 0.28 a	2.14 ± 0.08 b	***	Fruity, waxy
V32	Ethyl hexadecanoate	Ester	37.768	1974	1975	1.59 ± 0.15 a	0.61 ± 0.10 b	0.58 ± 0.04 b	***	Waxy, creamy, milky, oily
	TOTAL					98.64 a	49.44 b	25.62 c	***	

CF = Chemical Family; RT = Retention Time; KI = Kovats Index; EXP = Experimental; LIT = Literature; ^†^ *** significant at *p* < 0.001. ^‡^ Values (mean of 3 replications) followed by the same letter within the same row were not significantly different (*p* > 0.05) according to Tukey’s least significant difference test. **^¥^** Odour descriptors of the volatile compounds.

## Data Availability

The original contributions presented in the study are included in the article, further inquiries can be directed to the corresponding author.
